# The Causes and Treatment of Diarrhœa—IV

**Published:** 1908-11-21

**Authors:** 


### Medicine.

### THE CAUSES AND TREATMENT OF DIARRHOEA—IV.

TREATMENT (*Continued*).

(2). The drug that above all others best evacuates intestinal irritants is castor oil, and in none too small a dose. There is little or no need to discuss any other aperient, for none other is so satisfactory in this emergency. There will be two difficulties in the way of giving it: first, the patient's parents or friends may object to the use of an aperient when there is already such extreme diarrhœa; secondly, the associated vomiting may render it difficult for the castor oil to be swallowed and retained. The first of these difficulties will usually be removed by a few words of explanation; the second may be overcome by giving to a full grown person either half a drachm of laudanum or half an ounce of brandy in water, or both, along with the castor oil. Unfortunately there is no hypodermic purgative known, so that if castor oil fails, evacuant enemata are likely to be the next resource. Having given the aperient forthwith, the question of whether it should be repeated upon the following day or not can only be decided upon the merits of each case. As a rule no large dose of it is required after the first; but some authorities like to continue with drachm or two drachm doses of Mistura Olei Ricini B.P. three or four times a day as long as the stools are at all offensive.

(3). To prevent micro-organisms in the bowel receiving fresh pabulum constitutes one of the very most important of all the principles in the treatment of severe and acute diarrhœa. The symptoms are due to the products of organisms; starving the latter diminishes their products and helps to cure the patient. The difficulty is that to starve the organisms one must starve the patient too. Unkind though this may seem to be, it is essential. No food whatever should be given for the first four and twenty hours. Barley water, imperial drink, soda water, or plain water may be allowed *ad libitum*, but nothing which contains carbohydrate, proteid, or fat, upon which the bacteria could grow, should be given even to a child for the first day.

The principle thereafter is to give first of all such kinds of food as will be digested, and absorbed high up in the alimentary canal, leaving little or no residue by the time they reach the place where most of the bacteria are flourishing. Albumen water is the wisest thing to start with, in small quantities, gradually increased. The proteid in this will be rapidly and entirely absorbed. Whey, particularly white wine whey, is also excellent; and whereas beef-tea is little good, beef juice as it is prepared in many hospitals is very good. If cessation of the vomiting and diminution in the number and offensiveness of the stools permit of it, the next step would be to give one of the various prepared and partially digested or malted milks, much diluted to begin with, and in small quantities less diluted, and in larger quantities later on.

It may be several days before the patient has got back to a diet of ordinary milk and barley water, but there is much more harm done by trying to give "nourishment" quickly than there is by waiting fully long. If undigested food stuffs pass down to where the micro-organisms are growing, the latter will suddenly increase afresh and produce a serious relapse that might have been avoided if one were content to wait a little longer before being more liberal with the diet.

(4). It is clearly impossible in a living person to render the contents of the alimentary canal sufficiently antiseptic to actually kill the offending micro-organisms in it. It is not necessary to kill them, however. If only one can inhibit their rate of multiplication sufficiently, they will be carried away in the stools faster than they breed and a cure will soon result. Comparatively small amounts of antiseptics will serve to inhibit bacterial growth, and there can be little doubt of the benefits of giving glycerinum acidi carbolici ♏v., or $\beta$-Naphthol gr. x., or sodii sulphocarbolatis gr. xx., or essential oil of cinnamon ♏v. in capsules, three or four times a day, in cases of the kind under discussion.

(5.) In some cases of acute diarrhœa and vomiting or of ptomaine poisoning, a residual diarrhœa persists even after the original exciting cause has presumably been eliminated. It is likely that in these cases a certain amount of true enteritis or colitis has resulted. This will seldom be so severe, or so obstinate to treatment, as are the cases of chronic diarrhœa to be discussed immediately; but it may require remedies of the nature of bismuth oxycarbonate (gr. xx.), bismuth and morphia, tannic acid (gr. x.), tannigen (gr. x. to xx), hæmatoxylin, and so forth, the drug and the dose of it being decided entirely according to the merits of each case.

The treatment of other forms of diarrhœa need not be described in so much detail as the above. In cases of acute irritant poisoning, such as that due to arsenic, antimony, or corrosive sublimate, the essential points are—first, to take steps towards the elimination of as much of the poison as possible; secondly, to neutralise what remains; thirdly, to combat the patient's collapse; and, fourthly, if diarrhœa still continues, to treat it then upon the same lines as any other diarrhœa due to acute entero-colitis.

If fæcal accumulation and impaction is the cause of the diarrhœa it may be necessary to remove the mass piecemeal with the finger or by means of a spoon; in other cases, a pint of warm olive oil slowly injected will help to soften the fæces sufficiently to allow a part to come away in a soap and water enema an hour or two later; repetition of the process gradually emptying the bowel of the whole of the accumulated fæces.

If the diarrhœa is due to stenosis from venereal stricture, or from carcinoma, treatment becomes surgical; some cases are sufficiently palliated by dilation of the stricture by bougies, others require colotomy, and a few are capable of cure by excision.

We have recently discussed the dietetic treatment of mucous colitis, and we need not now repeat what we then said. There remain chronic cases and those in which the evacuations continue after the matters that have recently been irritating the bowel have been got rid of. Treatment in these cases resolves itself under four main headings—namely (1) dietetic, (2) by astringent remedies given by the mouth, (3) by lavage with astringent solutions per rectum, (4) by operation.

1. *Dietetic.*—In the relief of constipation it is usual to recommend fresh fruits, greens, oatmeal, marmalade, and so forth, in order that the undigested residue may produce abundant fæces, and hence more frequent defæcation. The converse holds true of chronic diarrhœa: the diet should contain as little irritating material incapable of digestion as possible; it may also be necessary to limit the quantity of liquid consumed, the ingestion of much water often making the diarrhœa worse. There are cases, however, in which the diarrhœa is much the same whatever the diet, and in these it is best not to make the patient's meals essentially different from those of healthy persons.

2. *By Astringent Remedies.*—In many cases of diarrhœa tincture of opium mxv., or Dover's powder gr. v. to x. are invaluable; morphia should not be employed, at any rate hypodermically, for fear that the habit may be contracted. An enema consisting of half a drachm of laudanum in two ounces of starch mucilage, and given with a small glass syringe, is well known in typhoid fever, and it is equally useful in some other cases of diarrhœa. The lead and opium pill is also invaluable. The old-fashioned chalk mixture, powdered ipecacuanha, catechu, krameria, kino, hæmatoxylin, tannic acid, and copper sulphate are all excellent remedies; the copper sulphate pill containing one-tenth to one-fifth grain of the salt, is very useful indeed in some cases of severe diarrhœa due to tubercular disease, though care must be taken not to increase the dose too rapidly for fear of vomiting.

3. *By Astringent Enemata.*—These are employed chiefly in chronic dysentery and in ulcerative colitis, the commonest solution being one of silver nitrate. The chief point to be remembered is that the enemata are extremely painful owing to the state of the bowel mucosa, and that, therefore, it is necessary to begin with very weak solutions, such as $\frac{1}{4}$ grain to the ounce, gradually increasing the strength until even 5 grains to the ounce can be borne. It may be impossible to use the injections at all to start with unless some tincture of opium is included in them. The opium may be gradually omitted as time goes on. In some cases the giving of the first few enemata may only be rendered possible by using a small quantity of a general anæsthetic.

4. *Operative measures.*—These consist essentially of either right inguinal colostomy or of appendicostomy, the first in order to prevent the large bowel being irritated by any fæces at all, the second in order to permit of the injection of astringent lotions from above downwards. In each case the operation is a temporary measure, performed in the hope that in six months' time or so the ulceration of the colon will be entirely healed, so that colostomy or appendicostomy openings can then be closed again.

